# Asymptomatic extreme troponin elevation preceding fatal clinically suspected sintilimab-associated myocarditis: a case report

**DOI:** 10.3389/fonc.2026.1889059

**Published:** 2026-08-19

**Authors:** Siqing Zhao, Zihan Song, Wenping Lu, Lei Chang

**Affiliations:** Guang’anmen Hospital, China Academy of Chinese Medical Sciences, Beijing, China

**Keywords:** biomarker, cardiotoxicity, gastric cancer, immune checkpoint inhibitor, myocarditis, sintilimab

## Abstract

**Background:**

Immune checkpoint inhibitors (ICIs) have been increasingly integrated into first-line systemic treatment strategies for advanced or metastatic gastroesophageal cancers. ICI-associated myocarditis is an uncommon but potentially fatal immune-related adverse event. Early recognition is particularly challenging when severe myocardial injury precedes typical cardiac symptoms.

**Case summary:**

A 71-year-old man with advanced gastric adenocarcinoma with liver, abdominal cavity, and retroperitoneal metastases received first-line oxaliplatin, S-1, and sintilimab on December 30, 2025. Baseline cardiac biomarkers before treatment were within normal limits, and baseline echocardiography showed preserved left ventricular systolic function without regional wall motion abnormality. He was rehospitalized for his second course of chemotherapy on January 23, 2026, without chest pain, palpitations, or dyspnea. Routine laboratory testing unexpectedly revealed extreme myocardial injury, with NT-proBNP 1263 pg/ml, cardiac troponin I (cTnI) >25, 000 ng/L, creatine kinase (CK) 5, 205 U/L, myoglobin (MYO) >1, 000 μg/L, and CK-MB mass 112.39 μg/L. Electrocardiography remained broadly similar to the baseline tracing of sinus rhythm with complete right bundle branch block, without a diagnostic acute ST-segment elevation pattern. Coronary angiography did not identify an acute culprit lesion sufficient to explain the biomarker elevation. Serial echocardiography showed new wall motion abnormality and progressive decline in left ventricular ejection fraction. Given the temporal relationship with PD-1 blockade, the absence of an angiographic culprit lesion, evolving myocardial dysfunction, and marked cardiac biomarker elevation, a clinical diagnosis of probable sintilimab-associated myocarditis was made. Intravenous methylprednisolone, intravenous immunoglobulin, and intensive supportive treatment were administered. However, myocardial biomarkers remained markedly abnormal, and overt cardiac symptoms emerged only later in the course, followed by hemodynamic instability and terminal circulatory collapse. The patient died of fulminant myocarditis with cardiogenic shock on February 1, 2026.

**Conclusion:**

This case illustrates a biomarker-first presentation of probable ICI-associated myocarditis, in which catastrophic myocardial injury was detected before typical cardiac symptoms became apparent. Absence of chest pain or dyspnea should not reassure clinicians when marked troponin elevation occurs shortly after ICI exposure.

## Introduction

Immune checkpoint inhibitors (ICIs) have reshaped the treatment landscape of many malignancies and are now incorporated into first-line treatment strategies for selected patients with advanced or metastatic gastric cancer, typically in combination with chemotherapy (1). Sintilimab, a programmed cell death protein 1 (PD-1) inhibitor, has shown clinically meaningful efficacy in combination treatment for advanced gastric or gastroesophageal junction adenocarcinoma (2). As the use of these agents expands, immune-related adverse events involving critical organs are increasingly recognized (3).

Among cardiovascular toxicities, ICI-associated myocarditis is uncommon but carries substantial mortality (4). Its clinical presentation spans a broad spectrum, ranging from asymptomatic cardiac biomarker elevation to conduction abnormalities, malignant arrhythmias, heart failure, cardiogenic shock, and sudden death (5). Early recognition remains difficult because some patients present without typical cardiac symptoms, even when myocardial injury is already severe (6).

Although fatal ICI-associated myocarditis has been previously reported (7), the value of the present case lies not in novelty of association, but in its striking presentation pattern: severe myocardial injury was first detected by routine biomarker testing while the patient remained free of chest pain, palpitations, and dyspnea. Here, we report a fatal case of probable sintilimab-associated myocarditis in a patient with advanced gastric adenocarcinoma, highlighting the dissociation between symptom burden and disease severity and the potential importance of early biomarker surveillance after ICI exposure.

## Case presentation

A 71-year-old man was diagnosed with advanced gastric adenocarcinoma with liver, abdominal cavity, and retroperitoneal metastases. He had no known family history of cardiomyopathy, premature coronary artery disease, sudden cardiac death, or inherited arrhythmia. His medical history was notable for hypertension, for which he was taking amlodipine besylate 2.5 mg once daily. He was not receiving a beta-blocker, angiotensin-converting enzyme inhibitor, angiotensin receptor blocker, mineralocorticoid receptor antagonist, sodium–glucose cotransporter 2 inhibitor, statin, or antiplatelet therapy before antitumor treatment. He denied any smoking history. His height was 166 cm and weight was 72 kg, corresponding to a body mass index of 26.13 kg/m², consistent with overweight rather than obesity.

Baseline cardiac assessment before sintilimab exposure was available. Electrocardiography (ECG) showed sinus rhythm with complete right bundle branch block (RBBB). Baseline cardiac biomarker testing performed in mid-December 2025 showed no evidence of myocardial injury, including cardiac troponin I (cTnI) 6.00 ng/L, myoglobin (MYO) 36.35 μg/L, CK-MB mass 0.39 μg/L, creatine kinase (CK) 49 U/L, and CK-MB activity 9 U/L. Baseline BNP was not available. And baseline transthoracic echocardiography on December 19, 2025 demonstrated normal chamber dimensions, no regional wall motion abnormality, preserved left ventricular systolic function, mild aortic stenosis, mild mitral regurgitation, and mild tricuspid regurgitation. Because the patient was initially diagnosed at an advanced/metastatic stage and had not received prior systemic antitumor therapy, oxaliplatin plus S-1 combined with sintilimab was administered as first-line systemic treatment. He had no previous exposure to fluoropyrimidines, platinum agents, immunotherapy, thoracic radiotherapy, or anthracyclines before this regimen. On December 30, 2025, the patient received the therapy. There were no documented acute cardiac symptoms during the first treatment course.

He was rehospitalized on January 23, 2026 for the planned second treatment cycle. At that time, he denied chest pain, chest tightness, palpitations, dyspnea, syncope, or new lower-limb edema. Physical examination did not reveal overt signs of acute heart failure. Body temperature was 36.2 °C, pulse rate 62 bpm, respiratory rate 18 breaths/min, and lung auscultation was clear. Cardiac rhythm was regular, and no pathologic murmur was detected. ECG obtained after sintilimab exposure continued to show sinus rhythm with RBBB, consistent with the baseline tracing, without a clear acute ST-segment elevation pattern.

**Figure 1:**
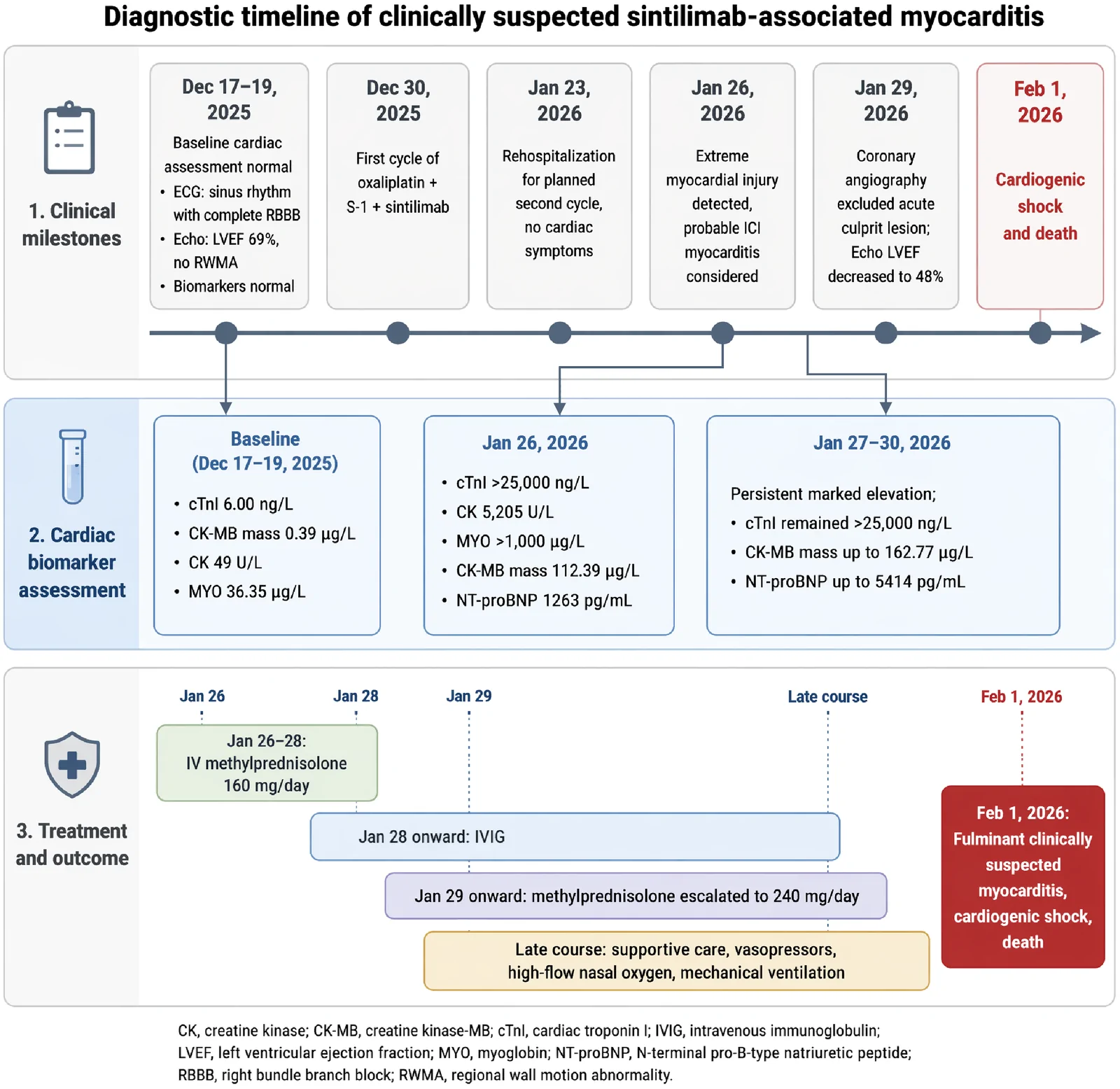
Clinical timeline from baseline cardiac assessment to fatal suspected sintilimab-associated myocarditis.

The overall clinical timeline from baseline cardiac assessment to terminal deterioration is summarized in **Figure 1**.

### Investigations

Routine laboratory testing after readmission unexpectedly showed marked myocardial injury. cTnI was >25, 000 ng/L, exceeding the upper analytical limit of the assay. CK was 5, 205 U/L, MYO was >1, 000 μg/L, and CK-MB mass was 112.39 μg/L. In parallel, inflammatory and immune-related laboratory abnormalities were also assessed, including CRP, IL-6, complete blood count, and lymphocyte subset profiling. Among these, IL-6 increased from 5.3 pg/mL at baseline to 16.55 pg/mL on January 26, 2026, while CRP did not show a marked concurrent rise at that time. These results were in sharp contrast to the normal baseline myocardial biomarker profile before sintilimab exposure. Serial myocardial biomarker results are summarized in **Table 1**, and additional laboratory findings are summarized in the **Supplementary Table 1**.

**Table 1:** Serial laboratory findings related to myocardial injury and cardiac stress.

Time point	cTnI ng/l	MYO μg/L	CK-MB mass μg/L	NT-proBNP pg/ml
Reference Range	0-53.53	0-110	0-5	0-125
12-17 9:42 (Baseline)	6.00	36.35	0.39	/
01-26 9:55	>25000	>1000	112.39	1263
01-26 10:11	>25000	>1000	134.56	/
01-26 16:39	>25000	>1000	101.27	/
01-27 06:28	>25000	>1000	143.18	1893
01-28 08:32	>25000	>1000	148.90	2120
01-29 08:25	>25000	>1000	160.42	3970
01-30 09:00	>25000	>1000	162.77	5414

**Figure 2:**
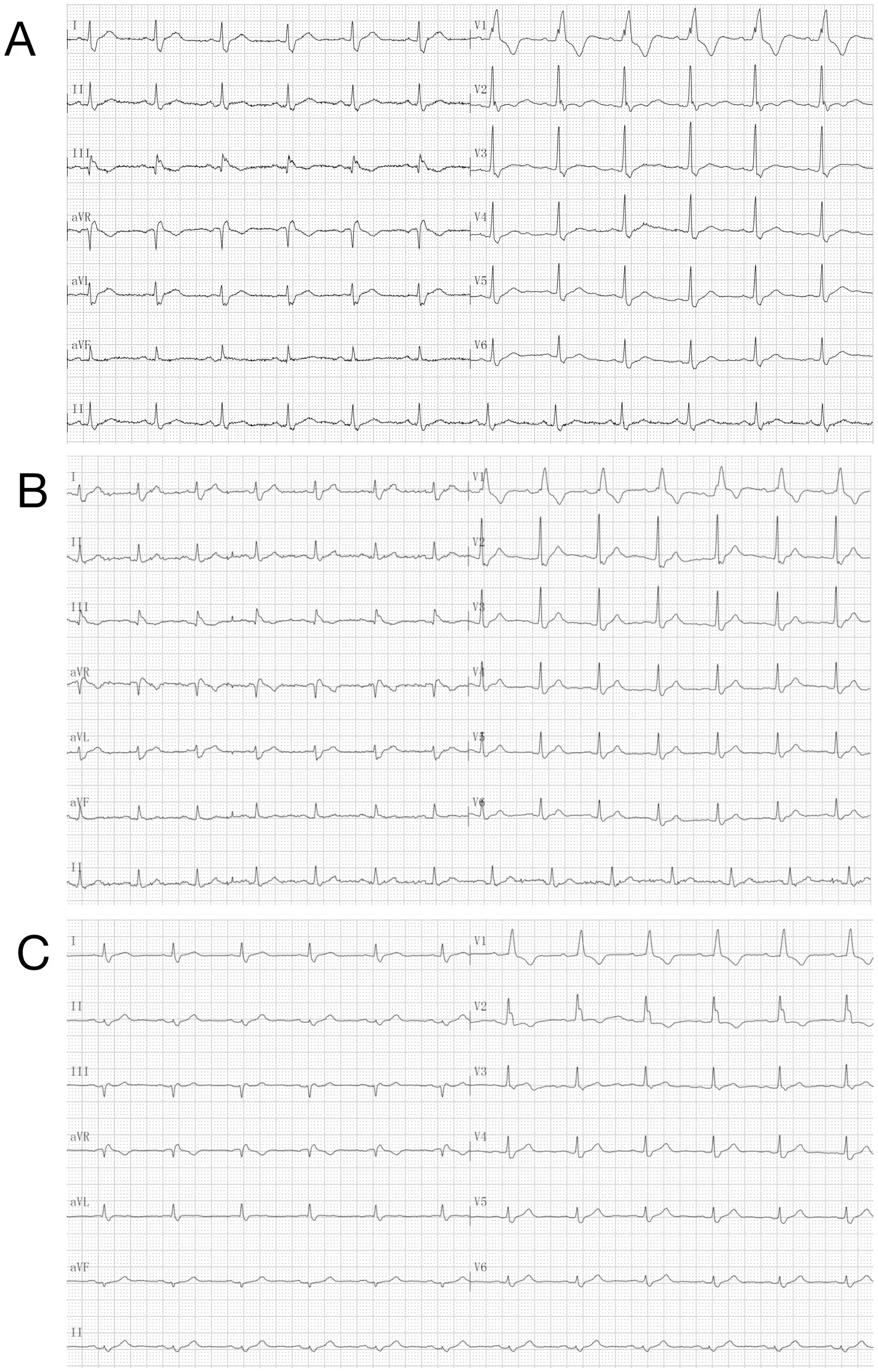
Serial 12-lead ECG from baseline to symptom onset. **(A)** Baseline (Dec 17, 2025): sinus rhythm with RBBB. **(B)** Admission (Jan 23, 2026): sinus rhythm with RBBB. **(C)** At biomarker elevation (Jan 26, 2026): sinus rhythm with left axis deviation and RBBB.

ECG after biomarker elevation again showed sinus rhythm with RBBBand new left axis deviation. The tracing was broadly similar to the baseline ECGand did not show diagnostic ST-segment elevation or dynamic ischemic changes proportional to the degree of biomarker elevation. Serial ECGs are shown in **Figure 2**. Because acute coronary syndrome could not initially be excluded, an emergency cardiology consultation was obtained. Repeat ECG on January 29, 2026 again showed sinus rhythm withRBBB, without a clear evolving ST-segment elevation pattern corresponding to the marked biomarker release. Bedside chest radiography suggested possible cardiomegaly and aortic atherosclerotic change.

Coronary angiography demonstrated a right-dominant system, with no significant stenosis in the left main artery, 20%–30% stenosis in the proximal left anterior descending artery with a myocardial bridge causing approximately 30% systolic luminal narrowing, no obvious stenosis in the circumflex artery, 70% stenosis in the proximal first obtuse marginal branch with TIMI 3 flow, and 50% stenosis at the ostium of the right coronary artery with TIMI 3 flow. No acute culprit occlusion was identified.

Serial transthoracic echocardiography showed new regional wall motion abnormality and progressive impairment of ventricular function after sintilimab exposure. These findings were not present on the baseline echocardiogram performed on December 19, 2025. Global longitudinal strain (GLS) was not available in this case. Serial echocardiographic findings are summarized in **Table 2**.

**Table 2:** Serial echocardiographic findings before and after sintilimab exposure.

Parameter	Baseline (2025-12-19)	2026-01-26	2026-01-29
Left atrial AP diameter, mm	35	41	38
LVIDd, mm	38	49	48
Right ventricular transverse diameter, mm	30	28	33
Aortic valve Vmax, cm/s	221	93	160
Mitral E/A, cm/s	99/85	66/98	64/87
Septal E′, cm/s	6.7	9.5	4
Lateral E′, cm/s	8.6	8.4	6
Septal E/E′	13.1	6.9	16.0
Lateral E/E′	12.7	7.9	10.7
LVEF, %	69	57	48
FS, %	34	30	24
TAPSE, mm	20	22.7	18
Chamber size	Normal	Left atrial enlargement	Left atrial enlargement
LV wall motion	No abnormality	Inferoposterior wall motion incoordination	Mildly reduced LV wall motion amplitude
Aortic valve	Mild aortic stenosis	Degenerative change	Degenerative change with mild aortic regurgitation
Mitral regurgitation	Mild	Not obvious on report	Mild
Tricuspid regurgitation	Mild	Not obvious on report	Mild
LV systolic/diastolic function	Preserved systolic function	LVEF preserved, but wall motion abnormality present	Reduced systolic and diastolic function
Other remarks	Normal chamber dimensions	Bedside study with limited assessment	Bedside study; irregular rhythm during examination

LV, left ventricular; LVEF, left ventricular ejection fraction; LVFS, left ventricular fractional shortening; E/e′, ratio of early mitral inflow velocity to early diastolic mitral annular velocity; TAPSE, tricuspid annular plane systolic excursion; SPAP, systolic pulmonary artery pressure.

We considered advanced imaging and histological confirmation; however, regrettably, the patient’s rapid clinical deterioration precluded further diagnostic investigations, including cardiac magnetic resonance imaging (CMR), positron emission tomography (PET), endomyocardial biopsy (EMB), and autopsy. Consequently, a definitive histopathological diagnosis could not be established.

### Differential diagnosis

A major diagnostic challenge in this case was distinguishing probable ICI-associated myocarditis from other causes of severe myocardial injury. Acute coronary syndrome was the most important initial alternative diagnosis because of the magnitude of troponin elevation. However, several findings argued against acute plaque rupture as the sole explanation: the absence of chest pain at initial detection, lack of diagnostic dynamic ST-segment elevation, absence of an acute culprit occlusion on coronary angiography, and progressive biventricular functional deterioration after recent sintilimab exposure. Although concomitant coronary atherosclerosis was present, the angiographic findings were more consistent with stable coronary artery disease than with a culprit lesion causing catastrophic myocardial necrosis.

Viral myocarditis could not be definitively excluded becauseCMR, EMB, autopsy, and complete viral testing were not performed. Nevertheless, no strong clinical evidence of an intercurrent viral syndrome was documented.

The possibility of chemotherapy-induced myocarditis was also carefully considered. Oxaliplatin and fluoropyrimidine-based agents may cause cardiac adverse events, most commonly ischemia, vasospasm, arrhythmia, or nonspecific myocardial injury. However, this patient had no previous fluoropyrimidine or platinum exposure, no typical acute ischemic chest pain syndrome temporally linked to drug infusion, no angiographic culprit lesion, and subsequently developed progressive ventricular dysfunction and cardiogenic shock in a pattern more compatible with immune-mediated myocarditis than with classic fluoropyrimidine- or platinum-related cardiotoxicity. Therefore, chemotherapy alone was considered less likely to fully explain the clinical course.

Another relevant consideration was possible overlap with ICI-associated myositis, because CK and myoglobin were markedly elevated. A myositis–myasthenia gravis–myocarditis (MMM) overlap syndrome could not be completely excluded; however, this possibility could not be fully evaluated in the absence of specific neuromuscular antibody testing, muscle imaging, electromyography, or histopathology. Importantly, dysphagia had already been present before the diagnosis of gastric cancer and before initiation of immunotherapy, and therefore could not be interpreted as a new immune-related neuromuscular symptom. In addition, no ptosis, diplopia, or clearly fluctuating limb weakness was documented before terminal deterioration.

Taking the overall picture together, the diagnosis remained clinically suspected/probable sintilimab-associated myocarditis, rather than histologically confirmed myocarditis.

### Treatment and clinical course

Once probable ICIs-associated myocarditis was suspected, sintilimab was permanently discontinued. At the time treatment was initiated, the patient had marked biomarker elevation but no overt cardiac symptoms, no definite new malignant arrhythmia, and no clearly diagnostic fulminant presentation at first recognition. Based on the *Current Chinese Society of Clinical Oncology (CSCO) Clinical Practice Guidelines in Cardio-Oncology (*8), the case was initially considered to resemble a lower-grade presentation driven primarily by biomarker abnormality. Therefore, intravenous methylprednisolone was initiated at 160 mg/day on January 26, 2026. Intravenous immunoglobulin was added at 200 mL/day on January 28. As biomarkers remained markedly abnormal and the clinical condition worsened, the methylprednisolone dose was escalated to 240 mg/day on January 29. Supportive therapy was provided according to the patient’s evolving clinical condition, including continuous cardiac monitoring, diuretics, dapagliflozin, and hepatoprotective agents. As cardiogenic shock developed, treatment for circulatory instability included vasopressors, high-flow nasal oxygen, and ultimately endotracheal intubation with mechanical ventilation. Despite corticosteroid-based immunosuppressive treatment and intravenous immunoglobulin, cardiac biomarkers did not show a clear downward trend, with serial cTnI measurements remaining above the upper limit of detection (>25, 000 ng/L), indicating ongoing myocardial injury.

### Outcome and follow-up

After the initial detection of myocardial injury, the patient remained at high risk despite limited early cardiac symptoms. Serial testing showed persistent myocardial biomarker abnormalities, and echocardiography demonstrated progressive ventricular dysfunction.

**Figure 3:**
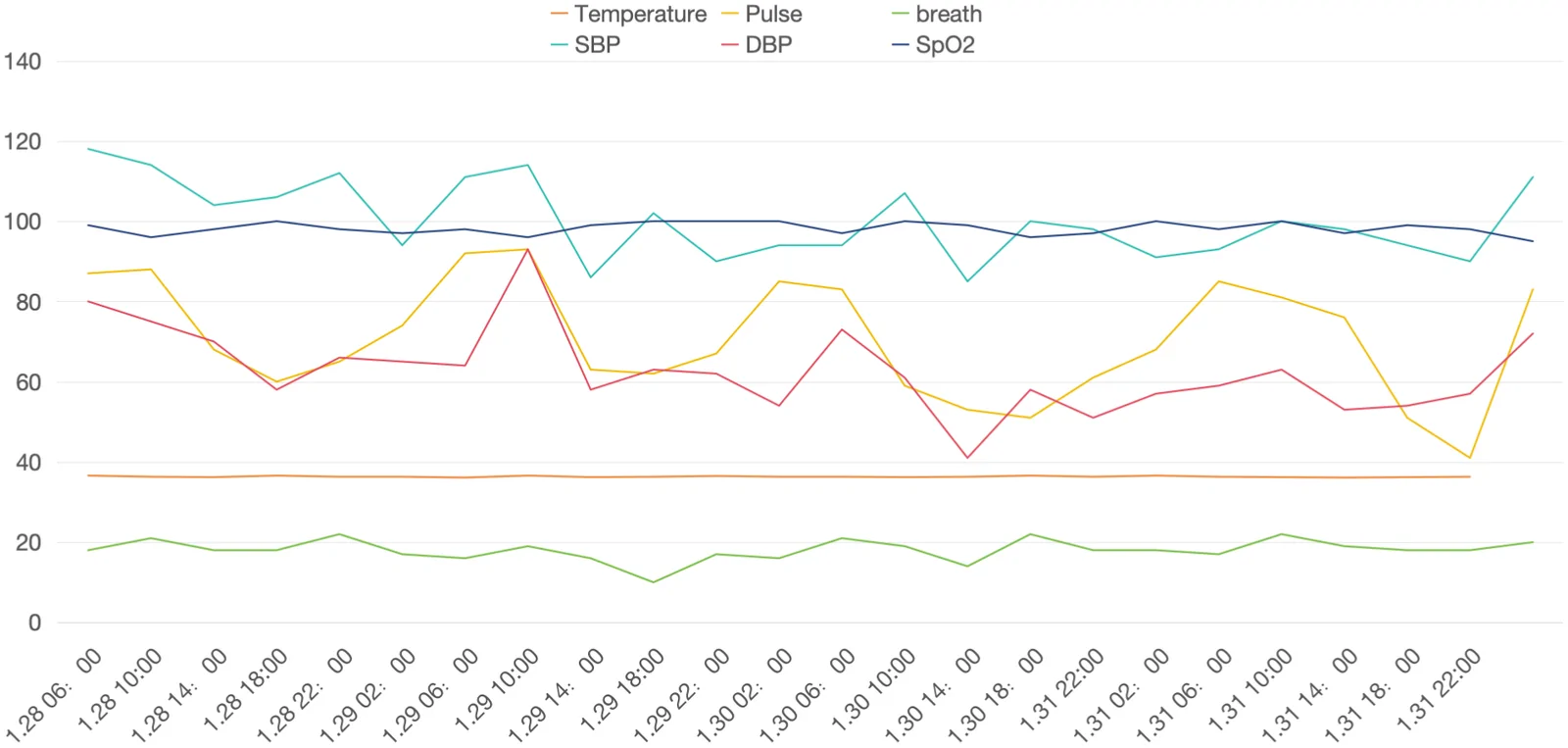
Vital sign trajectory preceding circulatory collapse.

The patient subsequently developed clinical deterioration with cardiogenic shock. Despite corticosteroids, intravenous immunoglobulin, and supportive care, there was no meaningful biochemical recovery and no sustained hemodynamic stabilization. He eventually died at February 1, 2026. The vital sign trajectory and terminal clinical course are summarized in **Figure 3**.

## Discussion

ICI-associated myocarditis is rare, but once clinically manifest it may be rapidly progressive and highly lethal (9). Fatal outcomes are especially concerning in patients who develop conduction abnormalities, ventricular arrhythmias, or cardiogenic shock (10). The present case falls within this fulminant spectrum, ultimately culminating in circulatory collapse and death.

The principal value of this report lies not in demonstrating a new drug-event association, but in illustrating a clinically important presentation pattern: life-threatening myocardial injury was first revealed by routine biomarker testing, while typical cardiac symptoms were still absent. Although presymptomatic troponin elevation has already been described in ICI-associated myocarditis, this case was notable for the extreme magnitude of cTnI elevation and the very rapid subsequent deterioration. At the time cTnI exceeded the upper analytical limit of the assay and CK, MYO, and CK-MB were all markedly elevated, the patient had no chest pain, palpitations, or dyspnea. Thus, symptom burden substantially underestimated disease severity. This presentation supports the concept that some ICI-associated myocarditis cases may initially appear clinically silent or smoldering despite carrying high biological risk (6).

This observation has practical implications. In routine care, chest pain, palpitations, dyspnea, or overt heart failure often trigger urgent cardiac evaluation. In contrast, this patient’s early complaints were dominated by dysphagia, fatigue, and gastrointestinal discomfort, creating a high risk that evolving cardiotoxicity could be underestimated. This case therefore supports the concept of a “biomarker-first” presentation of ICI-associated myocarditis, in which catastrophic myocardial injury may precede a recognizable cardiac syndrome.

The diagnosis in this case was necessarily clinical rather than histopathologic. Acute coronary syndrome was a legitimate early consideration given the magnitude of troponin elevation. However, coronary angiography did not identify an acute culprit lesion sufficient to account for the degree of biomarker release or the subsequent fulminant course. In the setting of recent sintilimab exposure, persistent severe biomarker abnormality, conduction disturbance, progressive ventricular dysfunction, and eventual cardiogenic shock, the overall picture strongly favored probable ICI-associated myocarditis (11). Nevertheless, because CMR, EMB, and autopsy were not performed, the diagnosis remained clinical rather than definitive.

Another important clinical lesson is the possible mismatch between symptoms and biological risk. *CSCO* (8) appropriately emphasize symptom assessment and grading, but this case suggests that a patient with minimal symptoms may still harbor catastrophic myocardial injury. In retrospect, the combination of cTnI above the assay limit, markedly elevated CK and MYO, persistent biochemical non-response, a and progressive impairment of ventricular function suggested a biologically aggressive phenotype from the outset (12). Put simply, symptom absence did not mean low risk.

The treatment course was also sobering. Corticosteroids and intravenous immunoglobulin were administered after probable myocarditis was recognized, but no meaningful biochemical recovery was observed and the patient subsequently deteriorated to cardiogenic shock. Although evidence remains limited, selected severe cases reported in the literature have required additional immunomodulatory strategies or advanced circulatory support beyond first-line corticosteroid therapy (13). Accordingly, persistent extreme troponin elevation after treatment initiation should prompt urgent reassessment of disease severity, intensified monitoring, renewed consideration of advanced diagnostic testing when feasible, and early multidisciplinary discussion regarding escalation of immunosuppression and hemodynamic support.

From a mechanistic perspective, checkpoint inhibitor-associated myocarditis is generally understood as a consequence of disrupted immune tolerance leading to T-cell-mediated myocardial injury (14). Experimental work supports an important role of the PD-1/PD-L1 axis in limiting cardiac immune damage, and its blockade may permit autoreactive inflammatory responses (15). Histopathologic observations further implicate inflammatory cell infiltration, including lymphocytes and macrophages, in myocardial injury (16). While this case does not offer direct mechanistic evidence, its fulminant course is consistent with a severe immune-mediated process.

Clinically, this case argues for greater vigilance during the early period after ICI initiation. Consensus-based recommendations support prompt interruption of immunotherapy and urgent evaluation when myocarditis is suspected (17). More proactive surveillance strategies, may help detect severe toxicity before overt hemodynamic decompensation, particularly in patients receiving combination treatment or with complex comorbidity profiles (18, 19). Emerging literature has also discussed the potential value of regular troponin monitoring during ICI therapy, although the optimal patient selection, frequency, and cost-effectiveness remain to be defined (20, 21). Our case further suggests that reliance on symptoms alone may be insufficient. In selected patients—particularly early after treatment initiation or in those receiving combination therapy—serial troponin measurement and ECG surveillance may help detect severe toxicity before overt hemodynamic decompensation. The value of such surveillance warrants further prospective evaluation.

A major strength of this case is the availability of pre-treatment cardiac baseline data. Before sintilimab initiation, myocardial injury biomarkers were within normal limits, and baseline echocardiography showed preserved systolic function without regional wall motion abnormality. These findings strengthen the inference that the subsequent extreme biomarker elevation and progressive ventricular dysfunction were treatment-emergent rather than pre-existing. In addition, complete RBBB was already present on baseline ECG, indicating that this conduction abnormality itself should not be misinterpreted as a newly developed immune-related event.

### Limitations

This report has several limitations. First, the diagnosis of ICI-associated myocarditis was clinical and remained probable because CMR, PET, EMB, and autopsy were not performed. Second, GLS was not available on echocardiography, which limited more sensitive assessment of early ventricular dysfunction. Third, although coronary angiography did not show an acute culprit lesion sufficient to explain the profound biomarker elevation, concomitant coronary atherosclerosis was present and may have contributed to myocardial vulnerability. Fourth, specific evaluation for overlap syndromes such as ICI-associated myositis or myasthenia gravis was incomplete; this is relevant because CK and myoglobin were markedly elevated. Fifth, complete exclusion of viral myocarditis was not possible. Sixth, the true troponin peak could not be determined because values exceeded the upper analytical limit of the assay. Finally, as a single fatal case without histopathologic confirmation, this report cannot establish causality or define the optimal treatment strategy, but it underscores the danger of relying on symptoms alone for early detection.

## Conclusion

In conclusion, this fatal case of probable sintilimab-associated myocarditis demonstrates that severe ICI-related myocardial injury may initially be clinically silent and first detected by routine biomarker testing. Extreme troponin elevation shortly after ICI exposure should prompt urgent evaluation for myocarditis even in the absence of chest pain or dyspnea. This case highlights a biomarker-first presentation pattern and reinforces the need for early cardio-oncology vigilance before irreversible hemodynamic collapse occurs.

## Data Availability

The original contributions presented in the study are included in the article/**Supplementary Material**. Further inquiries can be directed to the corresponding author.
