# Supplementary material for: Asymptomatic extreme troponin elevation preceding fatal clinically suspected sintilimab-associated myocarditis: a case report
Source: Front Oncol. 2026 Aug 19;16:1889059. doi: 10.3389/fonc.2026.1889059 (PMC13533704; doi:10.3389/fonc.2026.1889059)
Supplement: Supplementary file 1 [file DataSheet1.pdf]

	Indicator (Unit)	Reference Range	2025/12/18	2025/12/25	2026/1/26	2026/1/28	2026/1/27	2026/1/30
**Complete Blood Count +CRP**	WBC (10^9/L)	3.5 - 9.5	6.2	6.2	4.59			7.62
	RBC (10^12/L)	4.3 - 5.8	4.3	4.3	**4.13**			**4.02**
	HGB (g/L)	130 - 175	**128**	**128**	**120**			**120**
	HCT (%)	40 - 50	**37.9**	**37.9**	**36.6**			**36.9**
	PLT (10^9/L)	125 - 350	266	260	184			194
	LYMPH% (%)	20 - 50	**12.6**	**12.6**	**10.5**			**5.6**
	MONO% (%)	3-10	**10.5**	**10.5**	**11.8**			8.4
	NEUT% (%)	40 - 75	**76**	**76**	**75.3**			**86**
	LYMPH# (10^9/L)	1.1 - 3.2	**0.78**	**0.78**	**0.48**			**0.43**
	MONO# (10^9/L)	0.1 - 0.6	**0.65**	**0.65**	0.54			**0.64**
	NEUT# (10^9/L)	1.8 - 6.3	4.71	4.71	3.46			**6.55**
	RDW-SD (fL)	37 - 50	42.7	43.7	**51.2**			**59.4**
	RDW-CV (%)	11.5 - 14.6	13.3	13.5	**16.9**			**18.1**
	CRP (mg/L)	0 - 10	**10.84**	**10.94**	2.8			0.54
**Comprehensive biochemistry test**	TP (g/L)	65–85	**63.1**	**62.8**	**60.7**	**62.8**	**61.8**	67.8
	ALB (g/L)	40–55	**32.1**	**33.4**	**30**	**29.3**	**31.8**	**32.7**
	ALT (U/L)	9–50	**14.6**	7.7	**61.8**	**91.1**	**128.1**	**155.6**
	AST (U/L)	15–40	20.4	12.7	**301.4**	**336.6**	**355**	**342**
	Cr (umol/L)	59–104	65	69	65	63	66	75
	GLU (mmol/L)	3.9–6.1	4.13	3.88	4.1	4.68	4.97	**6.82**
	TCO2 (mmol/L)	22–29	26.6	26.6	26.6	28	25.9	27.7
	K (mmol/L)	3.5–5.3		4.49	4.16	4.55	4.86	4.86
	Na (mmol/L)	137–147		137.6	138.1	137	137.4	137.1
	Cl (mmol/L)	99–110		101.7	102.5	102.3	102.2	101.2
**blood clotting function**	PT (S)	10-14	12.5		12			11.5
	PT% (%)	70-150	104.3		116			129.6
	INR	0.8-1.5	0.99		0.95			0.91
	APTT (S)	23.3-32.5	26.5		23.4			**21.7**
	FIB (g/L)	2-4	3.78		**4.55**			2.97
	TT (S)	13-18	18		15.3			17
	FDP (mg/L)	0-5	2.5		4.5			**6.3**

	D-Dimer (mg/L)	0-0.55	0.4	**1.65**	**2.68**
	AT-III (%)	70-130	90.2	85	96
Tumor marker	AFP (IU/mL)	0-5.8	1.69	2.12	
	CEA (ng/mL)	0-5	**9.42**	**5.14**	
	TPSA (ng/mL)	0-4	0.486	**0.455**	
	FPSA (ng/mL)	0-0.934	0.0735	**0.0972**	
	PSA-Ratio	-	0.15	0.21	
	CYFRA21-1 (ng/mL)	0-3.3	**4.46**	3.22	
	NSE (ng/mL)	0-16.3	9.52	**20.5**	
	CA72-4 (U/mL)	0-6.9	5.3	3.39	
	CA19-9 (U/mL)	0-34	3.1	**74**	
	SCC (ng/mL)	0.5-2.7	2.19	0.986	
	CA242 (U/mL)	0-20	**174.04**	**36.14**	
Cytokine	IL-1β (pg/mL)	≤12.3	2.03	2.11	
	IL-8 (pg/mL)	≤62	25.49	13.04	
	IL-10 (pg/mL)	≤9.1	2.87	8.04	
	IL-6 (pg/mL)	≤7	5.3	**16.55**	
	IL-17 (pg/mL)	≤19	5.16	3.77	
	IL-2 (pg/mL)	≤8.2	2.05	2.24	
	IL-4 (pg/mL)	≤11.5	1.81	1.81	
	IL-5 (pg/mL)	≤8.7	1.67	2.62	
	IL-12p70 (pg/mL)	≤8.4	1.72	1.88	
	IFN-γ (pg/mL)	≤16.2	2.88	3.68	
	IFN-α (pg/mL)	≤13.2	2.01	2.21	
	TNF-α (pg/mL)	≤8	2.31	6.11	
Lymphocyte subsets	CD3+ (%)	50.0-84.0	54.46	62.48	
	CD19+ (%)	5.0-18.0	**3.6**	**4.82**	
	CD16+CD56+ (%)	7.0-40.0	**41.31**	31.73	
	CD4+ (%)	27.0-51.0	34.53	44.64	
	CD8+ (%)	15.0-44.0	19.44	17.33	
	CD4/CD8	0.71-2.78	1.78	2.58	
	CD3+ Absolute (cells/μL)	955-2860	**541.74**	**318.57**	

	CD19+ Absolute (cells/μL)	90-560	35.81	**24.57**
	NK Absolute (cells/μL)	150-1100	410.94	**161.82**
	CD4+ Absolute (cells/μL)	550-1440	**343.54**	**227.63**
	CD8+ Absolute (cells/μL)	320-1250	**193.36**	**88.35**
**Refined T cell subsets**	Activation of total T cells	1.2-5.8	**17.7**	**20.2**
	Quiescent total T cells	55.4-78.9	**36.9**	**42.6**
	Activated Helper T Cells	2.4-9.6	**16.8**	**19.7**
	CD8 CD38 (Activated)	6.3-23.8	**56.3**	**56.3**
	CD8 CD45RA CD45RO-	32.4-57.4	**21.8**	**13.5**
	Naive CD4 T Cells	31.6-54.4	**7.3**	**8.6**
	Memory CD4 T Cells	45.6-68.4	**34.8**	**25.3**
	CD8 CD28 (Functional)	37.2-60.4	**71.2**	**66.3**
	Treg (Regulatory T Cells)	3.0-7.0	**7.4**	**10**
	CD4 CD25 (Percentage of CD4)	14.92-27.44	**47.2**	**60.8**
